# Fat intake and injury in female runners

**DOI:** 10.1186/1550-2783-5-1

**Published:** 2008-01-03

**Authors:** Kristen E Gerlach, Harold W Burton, Joan M Dorn, John J Leddy, Peter J Horvath

**Affiliations:** 1 Department of Physical Therapy, The College of St. Catherine, Minneapolis, MN, USA; 2 Department of Exercise and Nutrition Sciences, University at Buffalo, Buffalo, NY, USA; 3 Department of Social and Preventive Medicine, University at Buffalo, Buffalo, NY, USA; 4 Department of Orthopaedics, University at Buffalo, Buffalo, NY, USA

## Abstract

**Background:**

Our purpose was to determine the relationship between energy intake, energy availability, dietary fat and lower extremity injury in adult female runners. We hypothesized that runners who develop overuse running-related injuries have lower energy intakes, lower energy availability and lower fat intake compared to non-injured runners.

**Methods:**

Eighty-six female subjects, running a minimum of 20 miles/week, completed a food frequency questionnaire and informed us about injury incidence over the next year.

**Results:**

Injured runners had significantly lower intakes of total fat (63 ± 20 vs. 80 ± 50 g/d) and percentage of kilocalories from fat (27 ± 5 vs. 30 ± 8 %) compared with non-injured runners. A logistic regression analysis found that fat intake was the best dietary predictor, correctly identifying 64% of future injuries. Lower energy intake and lower energy availability approached, but did not reach, a significant association with overuse injury in this study.

**Conclusion:**

Fat intake is likely associated with injury risk in female runners. By documenting these associations, better strategies can be developed to reduce running injuries in women.

## Background

The increased popularity of recreational and competitive running among females has led to an increased annual incidence of running-related injuries [1]. These injuries result from a complex interaction of female physiology with numerous risk factors that include sudden increases in training volume or intensity and a history of previous running injuries [1]. With the exception of calcium intake and incidence of stress fractures, though, nutrition as a contributing factor to running injuries has not been well-studied [2-4].

Although not yet found to be associated with overuse injury, numerous studies have reported a large negative energy balance in female runners [5-8], with some controversy as to which factor is most important – overestimation of energy expenditure, underestimation of energy intake, enhanced metabolic efficiency, or a true chronic deficiency that results in hormone abnormalities and altered reproductive function [8]. At least one author [8] has argued that the phenomenon of chronic energy deficiency is very real and manifested by a spectrum of reproductive hormone abnormalities that range from the less severe ovarian dysfunctions of follicular/luteal suppression and anovulation to the more severe amenorrhea. This author also states that, while some researchers consider this phenomenon a metabolic efficiency, it really is a pathological adaptation to scarce energy supplies that may interfere with crucial physiological processes, including reproduction, tissue maintenance, bone formation, and immunity [8].

Lending support to the idea that chronic negative energy balance is linked to altered reproductive function is recent work showing that an energy availability (defined as dietary energy intake minus exercise energy expenditure) below 30 kilocalories per kilogram of fat-free mass per day (kcal/kgFFM/d) is associated with menstrual and ovarian dysfunction due to reduction in pulsatile release of luteinizing hormone from the pituitary [9,10]. Such dysfunction can lead to decreased levels of serum estrogen, altered calcium metabolism and bone loss, similar to that seen in postmenopausal women. An energy availability between 20–30 kcal/kgFFM/d has also been found to impair bone formation due to a sharp decline in the osteocalcin available for bone matrix mineralization [11], as well as possible estrogen-independent mechanisms that include a disruption, also induced by low energy availability, of metabolic hormones such as triiodothyronine (T3) and insulin-like growth factor (IGF)-I. In addition, disordered or restrictive eating, menstrual irregularities, and inadequate calcium intake have been associated with increased risk of stress fractures in female runners [2,4,12]. However, no studies to date have examined the effect of energy availability on other types of overuse running injuries.

In addition to the effect of total energy intake on reproductive health, endurance performance and injury risk, fat consumption is of particular interest. Previous studies have shown a relationship between fat intake and amenorrhea [7] and endurance [13,14]. In the latter study, runners showed a reduced endurance performance while on a low fat diet (16%), compared to medium (31%) and high (44%) fat diets [14]. Interestingly, as fat intake increased, so did total energy intake [13]. At least 2 previous studies have found an association between fat intake and stress fractures. Bennell reported a lower fat diet was predictive of stress fractures in female track and field athletes, but total energy intake was not [2]. Wiita and Stombaugh [15] followed elite adolescent runners for a period of 3 years and found that both mean energy intake and fat intake decreased over the follow-up period while stress fracture incidence increased. Neither of these studies examined energy availability though, nor other types of overuse running injuries.

To further elucidate the relationship between energy intake, energy availability, fat intake and running injuries, we looked for relationships among diets of female runners and injury incidence over a one-year period. We hypothesized that runners who develop overuse running-related injuries have lower energy intakes, lower energy availability and lower fat intake compared with non-injured runners. A secondary aim of the study was to document the diets of competitive female runners and compare nutrient intake to the most recent DRI [16-20].

## Methods

This study was part of a larger multi-factorial analysis of risk factors for lower extremity injury in female runners [21]. Runners were recruited through flyers at local races, college campuses, and health clubs; advertisements in local running newsletters and web sites; and by e-mail to area running clubs. Ninety healthy adult female runners, aged 18 – 53 and running a minimum of 20 miles/week, participated. Most subjects were competitive at the local and regional running levels and a few were national caliber athletes. Those with a current injury to the lower extremities and/or low back or any who were pregnant within the past year were excluded. This study received approval from the University at Buffalo Human Subjects Review Board and all subjects provided informed, written consent.

After initial screening, subjects who met inclusion criteria were mailed the consent form and a questionnaire that asked about medical, menstrual, and training history, as well as history of previous running injury. With the exception of the prospective injury follow-up questionnaires, all measurements and questionnaires were completed at the time of study enrolment. These questionnaires included the Nutritionist 5 Food Frequency Questionnaire (FFQ) (First DataBank, San Bruno, CA/Axxya Systems, Stafford, TX), which asked them to quantify the frequency of intake of standardized serving sizes of 114 food items in a one-year period. The Nutritionist 5 FFQ is based on the "Block 98" FFQ originally developed by Block et al. [22,23]. Each subject was instructed by a registered dietician how to correctly complete the questionnaire. In addition, each runner completed the Eating Attitudes Test (EAT), a 40-item inventory which measures abnormal attitudes toward food and in which a higher score predicts the likelihood of disordered eating behaviors [24]. Subjects also received 24-hour activity logs and the Minnesota Leisure Time Physical Activity Questionnaire, which evaluates average daily, weekly or monthly energy expenditure in leisure time physical activity over a one-year period [25]. Both the activity log and leisure time questionnaire were returned with the FFQ and EAT during a visit to the laboratory, at which time each form was individually reviewed with the subject to ensure accuracy and completeness. During this visit, each subject's height and weight were recorded and Body Mass Index (BMI) was calculated as kg/m^2^. Percent body fat was calculated using skin folds [26]. Subjects underwent additional assessments for the multi-factorial analysis including measures of flexibility, VO2max, and pre-and post-fatigue balance and ground reaction forces [21].

Subjects were contacted every three months for one year and asked about the frequency, intensity, and duration of their running; about any changes in their health or menstrual status; and to describe the occurrence of any running-related injuries. A "running-related" injury was defined as any musculoskeletal injury to the low back or lower extremities of an overuse nature that occurred as a result of participation in running with one or more of the following consequences: reduction in the amount or level of running (including a decrease in the usual distance, frequency, or speed of training runs or races), a need for medical advice or treatment, or adverse social or economic effects (such as the inability to go to work due to the injury) [27]. All existing medical records were obtained to confirm injury diagnosis.

The FFQs were analyzed using Nutritionist Pro software (First DataBank, San Bruno, CA). Estimated energy expenditure (EEE) was calculated from the 24-hour activity logs using each subject's age and weight to estimate resting metabolic rate (RMR) and then multiplying RMR by weighted activity factors based on the duration and intensity of the activities reported in the log [28]. Exercise energy expenditure (XEE) was calculated using reference formulas and intensity codes from the Minnesota Leisure Activity Questionnaire [25] and expressed in units of kcal/kgFFM/d. The Minnesota Leisure Time Physical Activity Questionnaire has been studied extensively in reliability and validation studies of a variety of populations [25,28]. Energy balance was calculated as energy intake (from FFQ) minus EEE (from activity log). Energy availability was calculated as energy intake minus XEE (from Minnesota Leisure) in normalized units of kcal/kgFFM/d.

Descriptive statistics (methods of central tendency and variation) were computed for subject characteristics, as well as total energy intake, EEE, XEE, energy balance, energy availability and intake of the following nutrients: carbohydrates, fat, protein, vitamins A, B_6_, B_12_, C, D, E, K, magnesium, calcium, iron, zinc, copper, and fiber using SPSS version 8.0 (Chicago, IL) and NCSS 2000 (Kaysville, UT). All data sets were examined to ensure they were normally distributed and once normality was established, one-way ANOVAs comparing injured and non-injured runners were run. P values for the variables of energy intake, energy balance, energy availability, and fat intake are reported as single-sided since the direction of interest was motivated by findings of previous studies [2,8,9,11]. P values for the other variables are reported as two-sided. In addition, one-sample t-tests were used to compare runner mean nutrient values with the DRI value when the mean runner value was below the DRI.

Dietary variables that approached (p < 0.20) or achieved significance (p < 0.05) in the univariate analyses were entered into a forward, stepwise multiple logistic regression in order to build a multivariate model with a minimal number of predictive variables. Percentage of fat in the diet was converted into a dichotomous variable for purposes of computing odds ratios for injury if dietary fat was below the commonly recommended 30% of total energy intake. The alpha level was set at 0.05 for all analyses.

## Results

Eighty-six out of the original ninety subjects were included in the FFQ and injury data analysis. Subject characteristics are listed in Table 1.

**Table 1:** Subject characteristics for total sample, runners who sustained an injury during follow-up, and non-injured runners.

	Total Sample (n = 86)	Injured (n = 47)	Non-Injured (n = 39)	p value (injured vs. non-injured)
Age (y)	37 ± 9.2	36 ± 9.7	37 ± 8.8	.752
Height (cm)	164 ± 6.3	165 ± 5.8	163 ± 6.8	.342
Weight (kg)	59 ± 7.1	58 ± 6.4	59 ± 7.9	.485
BMI (kg/m^2^)	22 ± 2.4	22 ± 2.5	22 ± 2.2	.206
Body Fat (%)	19 ± 5.2	20 ± 5.5	19 ± 4.8	.445
Miles Run per Week	30 ± 9.1	31 ± 10.5	29 ± 7.2	.366
EAT score	15 ± 9.2	15 ± 9.3	15 ± 9.2	.812
XEE (kcal/kgFFM/d)	13 ± 6.2	14 ± 6.9	12 ± 5.1	.301
*EEE (kcal/d)	3127 ± 474	3123 ± 420	3132 ± 541	.942

Values presented as mean ± SD.

* Energy Expenditure values based on a total n = 72 (40 injured and 32 non-injured)

### Injury Incidence

Forty-seven subjects (55 %) reported a running-related injury during the one-year follow-up period with the foot/ankle the most common site (40 % of injuries), followed by the knee (19 %) and the hip (16 %). Stress fractures/stress reactions, iliotibial band problems, and tendonitis were the most common injuries. Of the 47 subjects who reported injuries, 37 (approx 80%) sought medical advice and it was these records that were obtained. For the other 10 subjects who did not seek medical advice, medical records did not exist, but each subject was contacted to ensure that their injury met the definition of a running-related injury. Of these 10, 8 runners indicated that their running had been disrupted for 1–3 wks, 1 for less than 1 week, and 1 for 4–8 weeks by the injury. Injured and non-injured runners did not differ significantly with respect to age, miles run per week, height, weight, body mass index, percent body fat, EAT scores, XEE, or EEE (Table 1).

### Dietary Intakes

No significant differences were observed between injured and non-injured runners in the total intakes or percentages of carbohydrate and protein in the diet, nor in the intake of dietary fiber, magnesium, calcium, iron, zinc, copper, or vitamins B_6_, B_12_, C, or D. (Table 2). One-sided p-values for daily energy intake (kcal/d), energy balance (kcal/kgFFM/d), and energy availability (kcal/kgFFM/d) approached significance (p = 0.07 – 0.08, Table 2) with values of the injured runners lower than the non-injured group. Injured runners did, however, consume significantly (p < .05) less total fat and obtain a lower percentage of total calories from fat than non-injured runners and consumed significantly lower amounts of the fat soluble vitamins A and K (Table 2).

**Table 2:** Daily dietary intake of total sample, injured, and non-injured runners.

	Total Sample (n = 86)	Injured (n = 47)	Non-Injured (n = 39)	p value (injured vs. non-injured)	Goal	p value (injured vs. DRI)	p value (all runners vs. DRI)
Energy Intake (kcal/d)	2120 ± 861	2002 ± 547	2262 ± 1123	*.083*	2567–2807		<.005**
Energy Intake (kcal/kgFFM/d)	45 ± 18.2	43 ± 11.8	47 ± 23.7	*.138*			
Energy Balance (kcal/kgFFM/d)	-20 ± 17.5	-22 ± 13.1	-16 ± 21.6	*.073*			
Energy Availability (kcal/kgFFM/d)	32 ± 17.8	29 ± 13.3	35 ± 21.8	*.070*			
% Protein	16 ± 3.0	16 ± 2.6	16 ± 3.4	.824			
% Carbohydrates	54 ± 7.2	56 ± 6.5	53 ± 7.8	.111			
% Fat	29 ± 6.4	27 ± 5.1	30± 7.6	.021**			
Protein (g)	85 ± 38	81 ± 25	90 ± 49	.240	46		
Protein(g)/kg/day	2 ± 0.7	1 ± 0.4	2 ± 0.9	.296			
Carbohydrates (g)	296 ± 119	288 ± 90	305 ± 147	.507	130		
Fat (g)	71 ± 38	63 ± 20	80 ± 50	.016**			
Dietary Fiber (g)	29 ± 14	28 ± 12	30 ± 16	.560	25		
Vitamin A (RE)	2164 ± 1105	1948 ± 869	2424 ± 1300	.046**	700		
Vitamin B_6 _(mg)	3 ± 1.1	3 ± 0.8	3 ± 1.3	.222	1.3		
Vitamin B_12 _(mg)	8 ± 6.4	7 ± 3.5	9 ± 8.7	.123	2.4		
Vitamin C (mg)	195 ± 90	197 ± 91	192 ± 91	.812	75		
Vitamin D (mg)	5 ± 4.5	5 ± 3.1	5 ± 5.9	.475	5	.450	
Vitamin E (mg)	14 ± 8	13 ± 6	16 ± 10	.065	15	.015**	
Vitamin K (mg)	52 ± 31	45 ± 24	60 ± 35	.019**	90		<.005**
Magnesium (mg)	427 ± 178	401 ± 128	458 ± 222	.136	320		
Calcium (mg)	1149 ± 661	1111 ± 520	1194 ± 803	.567	1000		
Iron (mg)	21 ± 12	20 ± 8	23 ± 15	.134	18		
Zinc (mg)	14 ± 7	13 ± 4	15 ± 9	.105	8		
Copper (mg)	2 ± 0.7	2 ± 0.6	2 ± 0.8	.095	0.9		

Values are presented as mean ± SD.

Goal values are taken from the most recent US DRI for healthy females age 19 – 50.

Estimated energy requirement in kilocalories taken from most recent DRI for very active female, 30 years of age, 1.65 m in height with BMI between 18.5 – 24.99 kg/m^2^.

One-sided p values are indicated in italics.

** One-way ANOVA significant at p < 0.05

With few exceptions, average daily dietary intakes were above the most recent US Dietary Reference Intakes (DRI) established by the Food and Nutrition Board for healthy females aged 19 – 50 (Table 2) [16-20]. Deficiencies were observed for dietary vitamin K for all runners (p < 0.005) and vitamin E for runners who developed injuries (p < 0.015). Mean daily energy intake was also below the Food and Nutrition Board recommendation of 2567–2807 kcal/d for very active normal weight women (p < 0.005) [20]. Mean daily energy intake approximated only two-thirds of EEE, indicating a negative energy balance.

A logistic regression analysis revealed that daily fat intake in grams was the single best dietary predictor of injury and this single dietary variable model was as accurate as any model combining multiple dietary variables. The model successfully classified 64% of the subjects in this study as subsequently injured (1) or not injured (0): 1.356 (SE = 0.61, p = .026) – 0.017 (SE = .008, p = .044)*intake of fat grams. A computed value above 0.5 would round to 1 and predict injury and a computed value below 0.5 would round to 0 and predict no injury. As such, the model correctly classified 39/47 (83.0%) of injured athletes and 17/39 (43.6%) of non-injured athletes. Thus, 8/47 injured athletes (17.0%) were incorrectly classified as not injured (false negatives) and 22/39 (56.4%) of non-injured athletes were incorrectly predicted to become injured (false positives).

The percentage of fat in the diet was converted into a dichotomous variable in order to facilitate the clinical application of these statistics by those making practical dietary recommendations to female runners. Thirty percent was chosen as the cut point as it is commonly recommended that 30% of total energy intake come from fat. The odds ratio for having <30% fat in the diet and suffering a running injury was 2.5 (95% CI 1.03 – 6.00, p = 0.04). Actual ratio proportions for less than 30% of energy from fat: 32 injured/18 non-injured (1.78:1) and more than 30% of energy from fat: 15 injured/21 non-injured (0.71:1).

## Discussion

Over half the runners in this study sustained a running-related injury in the year following their initial assessment. These injured runners consumed a diet significantly lower in total fat and lower in percentage of total energy from fat. This finding agrees with two studies which both reported correlations between low fat diets and incidence of stress fracture risk in female runners [2,15]. Fat intake was the most useful dietary factor in predicting future injury using a logistic regression, which may be of some clinical value to sports nutritionists. Further, the odds ratios revealed that runners consuming less than the commonly recommended 30% of total calories from fat were 2.5 times as likely to sustain an injury compared with runners consuming 30% or more. Interestingly, the highest fat intake of the injured group was 35.8% of total energy. Nine runners in the non-injured group exceeded this (with values of 36 – 47%) and sports nutritionists may want to consider ~36% as a conservative minimum fat intake for avoiding injuries, as long as carbohydrate and protein needs are also met.

One of the limits of observational studies such as this is the inability to determine cause-and-effect relationships, as well as the presence of numerous uncontrolled variables that may confound the results. And while we attempted to account for the most important of these factors, weekly running mileage, certainly other confounding variables remain. As such, the mean differences in fat intake between the two groups were admittedly small and, as demonstrated by the prediction accuracy of the logistic regression, certainly far from the only cause of running-related injury. Fat intake may play a more important role in the development of certain types of injuries (i.e. stress fractures) or in runners where energy intake is already heavily compromised. The benefit of the increased fat intake found in this study may in fact lie with an increase in ad libitum energy intake similar to that seen in work by Horvath et al [13] and it was originally hypothesized that overuse injury would also be associated with lower energy intakes. The energy intake values approached, but did not reach, a significant difference between injured and non-injured runners in this study, possibly due to confounding variables mentioned above.

Other recent research has focused on the role of restrictive eating or dietary restraint in female runners, which often includes restricted fat intake, and at least one study found an association between increased levels of cognitive dietary restraint and stress fractures [29]. However, if lower fat intakes among runners who developed overuse injuries in this study were indicative of restrictive eating patterns, this was not revealed by the EAT scores, which also were not significantly different between groups. Low levels of fat consumption have been shown to compromise energy supplies and contribute to excessive fatigue while running [14], and while we have observed alterations in running stride with fatigue, including a decrease in the impact peak and loading rate of ground reaction forces [21], this has not been conclusively proven to alter injury risk.

Another intriguing explanation for the correlation between fat and injury involves polyunsaturated fatty acids (PUFA), which are known to play a role in inflammation [30]. Deficient intake of *n*-3 PUFA could theoretically contribute to an enhanced inflammatory response and increase injury severity and, in fact, injured runners did consume significantly less PUFA (13.3 ± 4.8 g/d vs. 17.2 ± 9.7 g/d, p = 0.016), although the distinction between *n*-6 and *n*-3 was not made. Future research might be directed toward the role of PUFA more closely, particularly in light of recent studies showing the clinical efficacy of *n*-3 PUFA supplementation in decreasing the inflammatory response in persons with rheumatoid arthritis, asthma, or acute respiratory distress syndrome, and after severe trauma [30].

We also observed that energy intake as estimated from the FFQ was only two-thirds of calculated EEE, despite the fact that the majority of runners reported a steady weight. Differences between energy intake and expenditure have been reported in other studies [5-8] and may result from suppression of resting metabolic rate (RMR) due to chronic undernutrition [31], making EEE calculations invalid in this population. Using the mean energy intake and mid-range of the Food & Nutrition Board recommendation [20], it appears the runners in this study potentially had a metabolic suppression factor of 21%, which approximates the suppression factors in another study [31]. Because of this potential metabolic suppression, energy availability may be a more valid measure of nutritional status because it indicates the amount of energy remaining for other body functions after accounting for exercise expenditure. As mentioned earlier, when energy availability falls below 30 kcal/kgFFM/d, the reproductive system is potentially impaired due to disruption of LH pulsatility [10]. Low energy availability also limits other crucial functions in athletes, including the tissue maintenance and repair needed following difficult workouts, which could lead to injury development. Ihle and Loucks [11] reported that bone formation is impaired with energy availability in the range of 20–30 kcal/kgFFM/d due both to a linear decline in Type I collagen formation, as well as an abrupt decline in osteocalcin concentration, which hinders bone matrix mineralization. More severe energy restriction (energy availability of 10 kcal/kgFF/d) resulted not only in disruption of bone resorption, but also the uncoupling of bone resorption from suppressed bone formation, which can lead to irreversible reductions in BMD and an increased susceptibility to fracture [11].

Interestingly, both groups of runners in this study were significantly below the energy availability of ~45 kcal/kgFFM/d (p < .005) recommended for runners and other endurance athletes [9] and the mean value of the injured was below the threshold of 30 kcal/kgFFM/d discussed in the preceding paragraph. Our hypothesis of a difference in energy availability was not supported, however, as the difference in energy availability between the injured and non-injured runners approached, but did not achieve significance (injured 29.4 ± 13.3 vs. uninjured 35.1 ± 21.8 kcal/kgFFM/d, one-sided p = 0.07). The variability in the non-injured group was larger than anticipated, however, and this resulted in a loss of statistical power that makes it impossible to rule out energy availability as a contributing factor to overuse injuries. Thus, future observational studies may want to examine energy availability and overuse injury with a larger sample size or focus on a more specific group of runners or type of injury. It is possible that energy intake and availability play a greater role in injury development in certain sub-groups of runners (younger runners, for example, who must have enough energy to support growth or in high mileage runners who must support additional tissue repair) or in certain types of injuries (i.e. stress fractures). For example, the 6 runners in this study who developed stress fractures had an energy availability of 23.7 ± 5.3 kcal/kgFFM/d, significantly below the 30 kcal/kgFFM/d (p = .034) threshold cited above, which suggests that the possible mechanisms of impaired bone formation may have contributed to these injuries. A longitudinal experiment, in which energy availabilities were tightly controlled for a lengthy period, would be necessary in order to determine the exact cause-and-effect relationship between energy availability and stress fractures or any other type of injury, although ethical constraints might limit the feasibility of such a study.

In addition to the differences noted in fat intake, differences in intakes of three other fat-soluble vitamins (K, E, and A) were also noted. Injured runners had significantly lower intakes of vitamin K. Vitamin K is crucial for both blood clotting and as a key co-enzyme in the carboxylation of osteocalcin. The latter is necessary in order for osteocalcin to bind calcium and bone mineralization to occur. While deficiencies of vitamin K based on coagulation system function might be rare, the new DRI suggests that the vitamin K intake required for healthy bones may be as high as 90 micrograms/d [19] to ensure adequate carboxylation of osteocalcin. The injured runners in this study had significantly lower intakes of vitamin K than non-injured runners and both were significantly below 90 micrograms/d (Table 2). Low vitamin K intakes have been associated with both decreased bone mineral density and increased fractures in the elderly [32] and while it is possible that Vitamin K deficiency played a role in the injury development of some runners, it was not a useful predictor of future injury.

Mean values for vitamin E intake in injured runners were significantly below the DRI. Vitamin E functions as an anti-oxidant and it remains unclear whether strenuous exercise increases the need for antioxidants in the diet [33], whether deficits in vitamin E affect performance, and if vitamin E supplementation reduces exercise-induced muscle damage [33]. Intake of another anti-oxidant, vitamin A, was significantly less in the injured group, but because the mean intake of both groups was nearly three times the DRI and vitamin A did not play a role in the predictive model, intake of this nutrient did not appear to play a role in running injury in these subjects.

In addition to the limitations of observational studies discussed earlier, subjects in this study were followed for only 1 year, which may not be a long enough time period to see the full effects of nutrition on injury development. The instrument used to measure nutrient intake, the FFQ, is not without limitations of its own and given the body-conscious mind-set prevalent in the sport of running and in our society, some runners may have underreported or otherwise attempted to alter the intake values. The FFQ does not assess supplement usage, and while general usage of supplements was not correlated with injury, actual intake of micronutrients might have been higher than reported in Table 2. The FFQ was selected over the more detailed three-and seven-day food records both for the ease of completion by the athletes and because the FFQ would be more reflective of subject intake over a longer period of time and less open to subject manipulation. However, future studies may want to follow a larger number of subjects for a longer period and include 3- or 7-day food records, as well as measurements of potential RMR suppression and biological measures of menstrual function.

## Conclusion

A lower daily fat intake and lower percentage of total energy from fat were associated with increased injury risk among competitive female runners. Lower energy intake and lower energy availability approached, but did not reach, a significant association with overuse injury in this study. By documenting these risk factors, it is hoped that future research will continue to investigate their role in injury development, thus leading to better strategies to predict and reduce running injuries in women.

## Competing interests

The author(s) declare that they have no competing interests.

## Authors' contributions

KG conceived the idea, participated in research design, subject interviews and follow-up, data collection and analysis, and manuscript preparation. HB participated in the development of the original idea, research design, data interpretation and manuscript preparation. JD participated in research design, data interpretation and manuscript preparation. JL participated in subject screening, interviews and follow-up, analysis of all diet records and medical reports, and manuscript preparation. PH participated in research design, collection and analysis of diet records, and statistical analysis and interpretation of data. All authors read and approved the final manuscript.

## References

[B1] van Mechelen W (1992). Running injuries. A review of the epidemiological literature. Sports medicine (Auckland, NZ.

[B2] Bennell KL, Malcolm SA, Thomas SA, Reid SJ, Brukner PD, Ebeling PR, Wark JD (1996). Risk factors for stress fractures in track and field athletes. A twelve-month prospective study. The American journal of sports medicine.

[B3] Cline AD, Jansen GR, Melby CL (1998). Stress fractures in female army recruits: implications of bone density, calcium intake, and exercise. Journal of the American College of Nutrition.

[B4] Myburgh KH, Hutchins J, Fataar AB, Hough SF, Noakes TD (1990). Low bone density is an etiologic factor for stress fractures in athletes. Annals of internal medicine.

[B5] Edwards JE, Lindeman AK, Mikesky AE, Stager JM (1993). Energy balance in highly trained female endurance runners. Medicine and science in sports and exercise.

[B6] Beidleman BA, Puhl JL, De Souza MJ (1995). Energy balance in female distance runners. The American journal of clinical nutrition.

[B7] Estok PJ, Rudy EB (1994). Nutrient intake of women runners and nonrunners. Health care for women international.

[B8] Loucks AB (2004). Energy balance and body composition in sports and exercise. Journal of sports sciences.

[B9] Loucks AB (2007). Low energy availability in the marathon and other endurance sports.. Sports Med.

[B10] Loucks AB, Thuma JR (2003). Luteinizing hormone pulsatility is disrupted at a threshold of energy availability in regularly menstruating women. The Journal of clinical endocrinology and metabolism.

[B11] Ihle R, Loucks AB (2004). Dose-response relationships between energy availability and bone turnover in young exercising women. J Bone Miner Res.

[B12] Tomten SE, Falch JA, Birkeland KI, Hemmersbach P, Hostmark AT (1998). Bone mineral density and menstrual irregularities. A comparative study on cortical and trabecular bone structures in runners with alleged normal eating behavior. International journal of sports medicine.

[B13] Horvath PJ, Eagen CK, Ryer-Calvin SD, Pendergast DR (2000). The effects of varying dietary fat on the nutrient intake in male and female runners. Journal of the American College of Nutrition.

[B14] Horvath PJ, Eagen CK, Fisher NM, Leddy JJ, Pendergast DR (2000). The effects of varying dietary fat on performance and metabolism in trained male and female runners. Journal of the American College of Nutrition.

[B15] Wiita BG, Stombaugh IA (1996). Nutrition knowledge, eating practices, and health of adolescent female runners: a 3-year longitudinal study. International journal of sport nutrition.

[B16] Standing Committee on the Scientific Evaluation of Dietary Reference Intakes FNB (1997). Dietary reference intakes for calcium, phosphorous, magnesium, vitamin D and fluoride.

[B17] Standing Committee on the Scientific Evaluation of Dietary Reference Intakes FNB (1998). Dietary reference intakes for thiamin, riboflavin, niacin, vitamin B6, folate, Vitamin B12, pantothenic acid, biotin, and choline.

[B18] Standing Committee on the Scientific Evaluation of Dietary Reference Intakes FNB (2000). Dietary reference intakes for vitamin C, vitamin E, selenium, and carotenoids.

[B19] Standing Committee on the Scientific Evaluation of Dietary Reference Intakes FNB (2001). Dietary intake references for vitamin A, vitamin K, arsenic, boron, chromium, copper, iodine, iron, manganese, molybdenum, nickel, vanadium, and zinc.

[B20] Standing Committee on the Scientific Evaluation of Dietary Reference Intakes FNB (2002). Dietary reference intakes for energy, carbohydrate, fiber, fat, fatty acids, cholesterol, protein, and amino acids.

[B21] Gerlach KE, White SC, Burton HW, Dorn JM, Leddy JJ, Horvath PJ (2005). Kinetic changes with fatigue and relationship to injury in female runners. Medicine and science in sports and exercise.

[B22] Block G, Hartman AM, Dresser CM, Carroll MD, Gannon J, Gardner L (1986). A data-based approach to diet questionnaire design and testing. American journal of epidemiology.

[B23] Block G, Woods M, Potosky A, Clifford C (1990). Validation of a self-administered diet history questionnaire using multiple diet records. Journal of clinical epidemiology.

[B24] Garner DM, Garfinkel PE (1979). The Eating Attitudes Test: an index of the symptoms of anorexia nervosa. Psychological medicine.

[B25] Taylor HL, Jacobs DR, Schucker B, Knudsen J, Leon AS, Debacker G (1978). A questionnaire for the assessment of leisure time physical activities. Journal of chronic diseases.

[B26] Jackson AS, Pollock ML, Ward A (1980). Generalized equations for predicting body density of women. Medicine and science in sports and exercise.

[B27] (1997). American College of Sports Medicine.
Minnesota leisure-time physical activity questionnaire. Medicine and science in sports and exercise.

[B28] (1985). World Health Organization Energy and Protein Requirements. Report of a Joint FAO/WHO/UNO Expert Consultation.

[B29] Guest NS, Barr SI (2005). Cognitive dietary restraint is associated with stress fractures in women runners. International journal of sport nutrition and exercise metabolism.

[B30] Calder PC (2002). Dietary modification of inflammation with lipids. The Proceedings of the Nutrition Society.

[B31] Myerson M (1991). Resting metabolic rate and energy balance in amenorrheic and eumenorrheic runners. Medicine and science in sports and exercise.

[B32] Bugel S (2003). Vitamin K and bone health. The Proceedings of the Nutrition Society.

[B33] Urso ML, Clarkson PM (2003). Oxidative stress, exercise, and antioxidant supplementation. Toxicology.

